# Zespół łamliwego Chromosomu x i Choroby *FMR1*-zależne - Objawy Kliniczne, Epidemiologia i Podłoże Molekularne Choroby

**DOI:** 10.34763/devperiodmed.20182201.1421

**Published:** 2018-04-12

**Authors:** Aleksandra Landowska, Sylwia Rzońca, Jerzy Bal, Monika Gos

**Affiliations:** 1 Zakład Genetyki Medycznej, Instytut Matki i Dziecka, Warszawa, Polska

**Keywords:** zespół łamliwego chromosomu X, FXS, FXPOI, FXTAS, *FMR1*, fragile X syndrome, FXS, FXPOI, FXTAS, *FMR1*

## Abstract

Zespół łamliwego chromosomu X (ang. Fragile X Syndrome, FXS) jest, po zespole Downa, najczęstszą dziedziczną przyczyną niepełnosprawności intelektualnej (NI). Stopień niepełnosprawności intelektualnej pacjentów z FXS jest różny i zależy głównie od płci. Zaburzeniom intelektualnym towarzyszą dodatkowe objawy takie jak opóźnienie rozwoju psychoruchowego, zaburzenia zachowania czy emocji.

W ponad 99% przypadków, choroba spowodowana jest występowaniem mutacji dynamicznej w genie FMR1 zlokalizowanym na chromosomie X. W wyniku ekspansji trójki nukleotydów CGG (>200 powtórzeń) dochodzi do zahamowania ekspresji genu, a tym samym znacznego obniżenia poziomu białka FMRP kodowanego przez gen FMR1. Ekspansja sekwencji CGG do zakresu premutacji (55-200 powtórzeń CGG) warunkuje wystąpienie nosicielstwa FXS i chorób FMR1-zależnych takich jak: przedwczesne wygasanie funkcji jajników związane z zespołem łamliwego chromosomu X (ang. Fragile X-associated Primary Ovarian Insufficiency, FXPOI) oraz zespołu drżenia i ataksji związanego z zespołem łamliwego chromosomu X (ang. Fragile X-associated Tremor/Ataxia Syndrome, FXTAS). W przypadku obu tych chorób objawy kliniczne występują dopiero u ludzi dorosłych.

Celem pracy jest przybliżenie aktualnej wiedzy dotyczącej podłoża molekularnego i epidemiologii zespołu łamliwego chromosomu X oraz innych chorób FMR1-zależnych.

## Abstract (en)

Fragile X syndrome (FXS) is the second most common inherited cause of intellectual disability (ID), after Down syndrome. The severity of ID in FXS patients varies and depends mainly on the patient’s sex. Besides intellectual disorders, additional symptoms, such as psychomotor delay, a specific behavioral phenotype, or emotional problems are present in FXS patients.

In over 99% of the cases, the disease is caused by the presence of a dynamic mutation in the FMR1 gene localized on the X chromosome. Due to the expansion of CGG nucleotides (over 200 repeats), FMR1 gene expression is decreased and results in the significant reduction of the FMRP protein level. The CGG expansion to premutation range (55-200 CGG repeats) is equivalent to the FXS carrier status and may cause FMR1-dependent disorders − fragile X-associated primary ovarian insufficiency (FXPOI) and fragile X-associated tremor/ataxia syndrome (FXTAS). In contrast to FXS, clinical symptoms of these diseases occur later in adulthood.

The aim of the article is to present the knowledge about the molecular background and epidemiology of fragile X syndrome and other FMR1-related disorders.

Niepełnosprawność intelektualna (NI) jest jednym z częstszych objawów obserwowanych w zespołach genetycznych. Definiuje się ją jako stan, ujawniający się przed ukończeniem 18 roku życia, w którym sprawność intelektualna jest istotnie niższa od przeciętnej (Tabela I). Towarzyszą temu deficyty w zdolnościach adaptacyjnych takich jak: porozumiewanie się, umiejętność samodzielnego dbania o swoje ciało (w tym samodzielne ubieranie się) i wykonywanie codziennych czynności domowych. Deficyty obejmują także sferę kontaktów interpersonalnych, umiejętności podejmowania decyzji (w tym: dotyczących samostanowienia o sobie), dbania o swoje zdrowie i bezpieczeństwo, radzenia sobie w szkole i/lub w pracy, a także samoorganizowania sobie wypoczynku. Przyczyną wystąpienia NI mogą być zarówno czynniki genetyczne (aberracje chromosomowe lub choroby monogenowe), jak również czynniki środowiskowe (infekcje w okresie prenatalnym, zaniedbania ze strony opiekunów).

**Tabela I:** Klasyfikacja niepełnosprawności intelektualnej wraz z kodami ICD-10. Table I. Intellectual disability classification with ICD-10 codes.

Typ niepełnosprawności intelektualnej (NI) *Type of intellectual disability (ID)*	Iloraz IQ zgodny z klasyfikacją Weschslera *IQ score according to Wechsler classification*	Kod ICD-10^*^ ICD-10 code^*^
NI lekka *ID mild*	69-55 (10-12 lat) *69-55 (10-12 years)*	F70
NI umiarkowana *ID moderate*	54-35 (6-9 lat) *54-35 (6-9 years)*	F71
NI znaczna *ID severe*	34-20 (3-6 lat) *34-20 (3-6 years)*	F72
NI głęboka *ID profund*	<20 (max. 3 r.ż.) *<20 (max. 3 years)*	F73

* w klasyfikacji ICD-10 występują jeszcze kody F78 i F79 oznaczające odpowiednio: Inne upośledzenie umysłowe i niepełnosprawność intelektualną, nieokreśloną.

* *In ICD-10 classification, there are also F78 and F79 codes meaning respectively: other mental retardation and not specified intellectual disability*.

Jedną z częstszych chorób genetycznie uwarunkowanych, w których przebiegu obserwowana jest niepełnosprawność intelektualna jest zespół łamliwego chromosomu X (ang. *Fragile X Syndrome*, FXS). Jest on także wymieniany jako jedna z częstszych genetycznych przyczyn autyzmu. Po raz pierwszy zespół łamliwego chromosomu X został opisany w 1943 roku przez Jamesa Purdona Martina i Julię Bell, dlatego czasem nazywany jest również zespołem Martina-Bell. Badacze w swojej pracy opisali rodzinę, w której niepełnosprawność intelektualna w stopniu znacznym została stwierdzona u 11 mężczyzn, podczas gdy kobiety nie wykazywały objawów choroby. Rodowód rodziny wskazywał na dziedziczenie choroby sprzężone z płcią. Potwierdziły to badania Herberta Lubsa, który w 1969 r. opublikował wyniki swoich spostrzeżeń, w których stwierdził, że u niektórych osób z NI, analiza kariotypu wskazuje na obecność charakterystycznego ,,przewężenia’’, zlokalizowanego blisko końca długiego ramienia chromosomu X, w pozycji Xq27.3 [1]. Obecność przewężenia sprawia, że chromosom X wygląda jakby był złamany i stąd właśnie wywodzi się nazwa choroby − zespół łamliwego chromosomu X. Dalsze badania z zastosowaniem klonowania pozycyjnego umożliwiły w 1991 r. opisanie genu *FMR1* (ang. *fragile mental retardation 1*), którego mutacje są przyczyną zespołu łamliwego chromosomu X [2].

Gen *FMR1* jest pierwszym genem, w którym zidentyfikowano mutację dynamiczną – znaczące zwiększenie liczby niestabilnych powtórzeń trójnukleotydowych CGG, jako przyczynę rozwoju choroby [3]. Ponadto, jak wykazały retrospektywne badania rodzin pacjentów z FXS, potwierdzone badaniami prospektywnymi, zwielokrotnienie powtórzeń CGG w genie *FMR1* może być także przyczyną wystąpienia przedwczesnego wygasania czynności jajników oraz zespołu drżenia i ataksji związanego z zespołem FXS (odpowiednio, FXPOI – *Fragile X-associated primary ovarian insufficiency* i FXTAS – *Fragile X-associated tremor/ataxia syndrome*).

W przypadku pacjentów z niepełnosprawnością intelektualną, badanie molekularne w kierunku zespołu łamliwego chromosomu X i identyfikacji mutacji dynamicznej w genie *FMR1* jest wykonywane rutynowo, zaraz po analizie kariotypu. Umożliwia ono nie tylko potwierdzenie rozpoznania klinicznego, lecz również jest wskazaniem do badania nosicielstwa zespołu w rodzinie, co pozwala na identyfikację potencjalnych pacjentów z FXPOI i FXTAS. Ponadto ostateczne potwierdzenie badaniem molekularnym rozpoznania klinicznego zespołów FXS, FXPOI i FXTAS ułatwia przyjęcie odpowiednich strategii w rehabilitacji i leczeniu. Umożliwia także uzyskanie odpowiedniej porady genetycznej dla całej rodziny pacjenta, dotyczącej ryzyka wystąpienia choroby u kolejnych osób z rodziny.

Celem pracy jest przedstawienie objawów klinicznych, epidemiologii i podłoża molekularnego zespołu łamliwego chromosomu X i chorób *FMR1*-zależnych.

## 1 Gen *FMR1* i jego produkt - Białko FMRP

Gen *FMR1*, którego mutacje odpowiedzialne są za wystąpienie objawów choroby jest genem silnie konserwowanym w toku ewolucji. Podobieństwo sekwencji aminokwasowej w grupie ssaków jest wysokie, przykładowo między człowiekiem a myszą wynosi 97%. Ponadto wzór ekspresji białka w poszczególnych tkankach jest podobny u wszystkich gatunków [4]. Ludzki gen *FMR1* składa się z 17 eksonów i koduje białko FMRP (ang. fragile X mental retardation protein) o wielkości 71 kDa [5].

Ssacze białko FMRP oraz jego paralogi autosomalne − FXR1P i FXR2P stanowią małą rodzinę białek wiążących cząsteczki RNA. Białka te wykazują wysokie podobieństwo sekwencji aminokwasowej (>60%), zaś geny je kodujące prawdopodobnie powstały wskutek duplikacji jednego genu (tzw. paralogi). Białka z rodziny FMRP zawierają trzy charakterystyczne, homologiczne domeny KH odpowiedzialne za wiązanie i regulację cząsteczek RNA (m.in. mRNA i rRNA). O tym jak istotną rolę pełnią te domeny w aktywności białka świadczy efekt mutacji punktowej p.Ile304Asn, która powoduje zmiany w obrębie drugiej domeny KH. Analiza struktury krystalicznej białka z mutacją wykazała, że jej obecność zakłóca prawidłowe fałdowanie domeny KH, co w konsekwencji zaburza wiązanie cząsteczek RNA przez białko i powoduje utratę jego funkcji [6].

Kolejną charakterystyczną domeną jest kaseta RGG – domena bogata w reszty argininy i glicyny, zlokalizowana w C-końcowej części białka, która wiąże cząsteczki RNA posiadające w swojej budowie charakterystyczną strukturę bogatą w nukleotydy guaninowe [7]. Białko FMRP zawiera również sygnał lokalizacji jądrowej, co sugeruje, że białko zlokalizowane głównie w cytoplazmie może przemieszczać się do jądra komórkowego, dokładny mechanizm i rola białka FMRP w obrębie jądra nie została jednak dobrze scharakteryzowana [8].

Białko FMRP ulega ekspresji głównie w komórkach nerwowych, zwłaszcza tych mających dużą liczbę synaps np. zlokalizowanych w obrębie hipokampu czy móżdżku. Białko stanowi część polirybosomu i jest zaangażowane w proces translacji białek odpowiedzialnych m.in. za plastyczność synaptyczną [9, 10]. Brak odpowiedniej regulacji syntezy specyficznych białek, szczególnie na poziomie synaps, skutkuje zmianami w tym procesie. Prowadzi to do zaburzeń procesów uczenia się i kształtowania pamięci, co jest przyczyną wystąpienia najważniejszych objawów choroby, czyli niepełnosprawności intelektualnej i osłabienia czynności poznawczych [5, 11, 12, 13].

## 2 Podłoże molekularne zespołu łamliwego chromosomu X

Jak wspomniano wcześniej, za znaczącą większość (>99%) przypadków zespołu łamliwego chromosomu X odpowiedzialna jest mutacja dynamiczna, związana ze zwielokrotnieniem liczby powtórzeń trójnukleotydowych CGG w regionie niekodującym (UTR, ang. *untranslated region*) na końcu 5’ genu *FMR1* (Rycina 1). Termin ,,mutacja dynamiczna’’ został wprowadzony w celu wyróżnienia unikatowej cechy jaką mają powtórzone sekwencje DNA, czyli możliwości spontanicznego wydłużania się (tzw. ekspansji). Udowodniono, że zwielokrotnienie liczby powtórzeń nukleotydowych może mieć istotne implikacje kliniczne – obecność mutacji dynamicznej w różnych genach stanowi przyczynę wystąpienia około 30 różnych chorób neurologicznych, neurodegeneracyjnych czy nerwowo-mięśniowych. Oprócz FXS, do grupy chorób wywołanych tym typem mutacji zaliczamy m.in. chorobę Huntingtona, dystro/ ę miotoniczną typu 1 i 2, wybrane ataksje rdzeniowo-móżdżkowe, niektóre rodzaje stwardnienia zanikowego bocznego czy otępienia czołowo-skroniowego. Wszystkie z wymienionych chorób, mimo że dotyczą różnych genów i różnych sekwencji powtórzonych, wykazują te same właściwości, np. dziedziczenie choroby i jej progresja uzależnione są od wyjściowej liczby powtórzeń nukleotydowych. Niestabilne powtórzenia wykazują również specyficzne właściwości strukturalne − udowodniono, że mogą one przybierać charakterystyczne formy przestrzenne np. kształt spinki do włosów. Struktury te sprzyjają „poślizgowi” polimerazy DNA, co zaburza procesy replikacji, rekombinacji, naprawy DNA i jednocześnie prowadzi do dalszego zwielokrotnienia powtórzeń sekwencji nukleotydowych [14].

Sekwencja nukleotydowa (CGG)n w genie *FMR1* wykazuje znaczną zmienność (tzw. polimor/ zm). Liczba powtórzeń tej trójki nukleotydowej może znajdować się w zakresie od 5 do ponad 750. Zgodnie z wytycznymi European Molecular Genetics Quality Network (EMQN) i American College of Medical Genetics (ACMG), w zależności od liczby powtórzeń CGG wyróżnia się trzy podstawowe typy alleli (Tabela II). Pierwsze z nich − allele prawidłowe mieszczą się w zakresie od 5 do 54 powtórzeń CGG i są zazwyczaj stabilnie przekazywane w kolejnych pokoleniach. W populacji polskiej najczęściej występują allele zawierające: 30 (20,17%), 29 (15,71%), 31 (8,21%), 23 (7,50%) i 20 (4,52%) powtórzeń sekwencji nukleotydowej CGG (wartości uzyskane z bazy danych osób z wykluczonym zespołem łamliwego chromosomu X, prowadzonej przez Zakład Genetyki Medycznej Instytutu Matki i Dziecka) [15]. Allele o wielkości od 40 do 44 powtórzeń spotyka się w populacji z częstością ok. 5%. Jeszcze rzadziej występują allele z zakresu tzw. ,,szarej strefy’’ (45-54 powtórzeń CGG). W populacji stwierdza się je z częstością 2% w zakresie 45-49 powtórzeń i 0,5% w przypadku alleli większych niż 50 powtórzeń [16]. Ponieważ allele z zakresu „szarej strefy” mogą być bardziej podatne na wydłużanie, to zgodnie z wytycznymi EMQN, identyfikacja takiego allelu u badanej osoby jest wskazaniem do analizy rodziny badanego w celu oceny stabilności jego dziedziczenia. Co ważne, w literaturze nie odnotowano przypadków wydłużenia się alleli z zakresu „szarej strefy” do pełnej mutacji w jednym pokoleniu [17].

**Ryc. 1:**
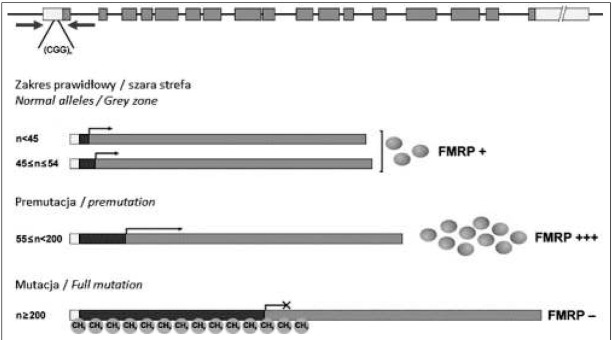
Schemat genu *FMR1* uwzględniający lokalizację sekwencji (CGG)n. Na rycinie przedstawiono poszczególne zakresy alleli w genie *FMR1* i ilość produkowanego białka FMRP. Fig. 1. The FMR1 gene scheme with CGG repeats. We presented all the categories of FMR1 alleles and the amount of the FMRP protein depending on allele length.

**Tabela II:** Typy alleli genu *FMR1* w zależności od liczby powtórzeń CGG. Table II. Types of FMR1 gene alleles depending on the number of CGG repeats.

**Typ allelu *Allele type***	**Liczba powtórzeń CGG *Number of CGG repeats***	**Fenotyp *Phenotype***	**Ryzyko ekspansji do pełnej mutacji *Risk of expansion to full mutation***
Prawidłowy *Normal*	<45	Prawidłowy *Normal*	Brak *None*
	45-54 (tzw. szara strefa) *45-54 („grey zone”)*	Prawidłowy *Normal*	Brak, ewentualnie do premutacji *None, optionally to premutation*
Premutacja *Premutation*	55-200	podwyższone ryzyko wystąpienia FXPOI u kobiet i FXTAS u obu płci *Increased risk of FXPOI for females and FXTAS in both sexes*	Wysokie, zwiększające się wraz ze wzrostem liczby powtórzeń CGG *High, increasing with the number of CGG repeats*
Mutacja *Full mutation*	>200	Zespół łamliwego chromosomu X - chłopcy i do 50% dziewczynek *Fragile X syndrome - males and up to 50% of females*	Sekwencja wysoce niestabilna *Highly unstable sequence*

Ponadto w przypadku genu *FMR1*, wyodrębnia się niestabilne allele z zakresu premutacji (55-200 powtórzeń) i allele z zakresu pełnej mutacji, którą stwierdza się przy liczbie powtórzeń powyżej 200. Dodatkowo, w przypadku obecności pełnej mutacji dochodzi do hypermetylacji regionu promotorowego genu, co prowadzi do wyciszenia jego ekspresji, a w konsekwencji do braku białka FMRP, co skutkuje zaburzeniami w postaci objawów zespołu łamliwego chromosomu X [18].

U osób będących nosicielami allelu z zakresu premutacji znacznie częściej dochodzi do ekspansji liczby powtórzeń sekwencji CGG, również do zakresu pełnej mutacji. Do zwiększenia liczby powtórzeń dochodzi najprawdopodobniej w komórkach rozrodczych, w trakcie procesu replikacji materiału genetycznego lub znacznie rzadziej, na początkowych etapach rozwoju zarodka. W przypadku genu *FMR1* mamy do czynienia z tzw. antycypacją matczyną – do ekspansji powtórzeń od premutacji do zakresu pełnej mutacji dochodzi w komórkach jajowych, w związku, z czym choroba przekazywana jest dzieciom przez matki nie wykazujące objawów choroby. Ojciec – nosiciel może przekazać allel z zakresu premutacji swoim córkom, które staną się w ten sposób nosicielkami choroby i będą miały blisko 50% ryzyko posiadania chorego potomstwa. Nie odnotowano przypadków ekspansji powtórzeń CGG do zakresu pełnej mutacji w linii ojcowskiej [19].

Allele z zakresu premutacji dość powszechnie występują w populacji (1/250-450 chłopców i 1/130-200 dziewczynek). W rodzinach obciążonych zespołem łamliwego chromosomu X częstość występowania premutacji jest wyższa niż pełnej mutacji. U nosicieli premutacji nie stwierdza się hypermetylacji genu *FMR1*, a tym samym nie jest zahamowana jego transkrypcja i synteza białka FMRP. Jak wspomniano wcześniej allele z zakresu premutacji są niestabilne i istnieje ryzyko ich ekspansji do pełnej mutacji w następnych pokoleniach. Ryzyko to jest zależne od liczby powtórzeń CGG i szacuje się, że w przypadku alleli >99 powtórzeń wzrasta ono do 100%. Sugeruje się także, że stabilność alleli może zależeć od obecności przerw w sekwencji (CGG)n w postaci sekwencji AGG. Zazwyczaj w prawidłowych, stabilnych allelach obecne są dwie sekwencje AGG (co około 10 powtórzeń CGG). W przypadku alleli niestabilnych (premutacji) przerw AGG jest znacznie mniej lub nie występują wcale.

Jak wspomniano wyżej pełną mutację w przypadku FXS stwierdza się przy wystąpieniu ponad 200 powtórzeń CGG. Sytuacja ta powoduje hypermetylację promotora oraz regionu 5’UTR genu *FMR1*, na skutek metylacji cytozyn zawartych w długim ciągu powtórzonej sekwencji. Metylacja tego regionu genu prowadzi do zahamowania jego ekspresji, a tym samym do zmniejszenia lub braku syntezy białka FMRP, co bezpośrednio przekłada się na ujawnienie objawów choroby. Przy wystąpieniu pełnej mutacji proces metylacji zachodzi zarówno u mężczyzn jak i u kobiet. Warto jednak podkreślić fakt, że w sytuacji, jeżeli u kobiet zmutowany gen będzie znajdował się na inaktywowanym chromosomie X, to pomimo stwierdzenia w badaniu molekularnym pełnej mutacji osoba taka, może nie wykazywać żadnych objawów choroby [20].

Ekspansja powtórzeń trójnukleotydowych CGG jest odpowiedzialna za wystąpienie >99% przypadków zespołu łamliwego chromosomu X. U 1% pacjentów opisywane są jednak inne zmiany w obrębie genu *FMR1*, takie jak delecja całego lub fragmentu genu czy mutacje punktowe, w tym mutacje typu missens (zmiana aminokwasu). Efektem tych mutacji będzie, podobnie jak w przypadku pełnej mutacji, brak (mężczyźni) lub znacząco obniżony poziom (kobiety) białka FMRP, a w przypadku zmian aminokwasowych, powstanie białka o zmienionej aktywności lub specyficzności względem cząsteczek, z którymi się łączy [21].

## 3 Objawy kliniczne zespołu łamliwego chromosomu X

Osoby z zespołem łamliwego chromosomu X wykazują szerokie spektrum objawów klinicznych, które w znacznej mierze uzależnione są od płci pacjenta oraz obecności zmian związanych z mutacją dynamiczną w genie *FMR1*. Podstawowym objawem zespołu łamliwego chromosomu X jest niepełnosprawność intelektualna, która stwierdzana jest u wszystkich chłopców z pełną mutacją i ma zazwyczaj stopień umiarkowany lub głęboki. U około 50% kobiet stwierdza się NI w stopniu lekkim. U większości pacjentów FXS niepełnosprawność intelektualna ujawnia się już w okresie wczesnodziecięcym [22].

Niepełnosprawności intelektualnej obserwowanej u pacjentów z FXS mogą towarzyszyć również takie objawy jak: opóźnienie rozwoju psychoruchowego, zwłaszcza w zakresie rozwoju mowy oraz zaburzenia zachowania i problemy emocjonalne [23]. U ok. 25-33% chorych stwierdza się zaburzenia ze spektrum autyzmu, które mogą ujawniać się w postaci ograniczonej zdolności do komunikacji ze społeczeństwem (unikanie kontaktu wzrokowego), braku koncentracji, impulsywności, agresji w stosunku do siebie i innych osób (dotyczy to szczególnie mężczyzn), nadpobudliwości, nadwrażliwości na bodźce dotykowe, słabej koordynacji ruchowej, zaburzeń lękowych czy występowania stereotypii ruchowych (np. trzepotanie rękoma czy kołysanie się) [24].

Poza niepełnosprawnością intelektualną i opóźnieniem rozwoju psychoruchowego, u osób z FXS występują także specyficzne cechy dysmorficzne – charakterystyczna jest długa, wąska twarz i odstające uszy. Objawy te występują u około 70% pacjentów i są silniej wyrażone u osób starszych. Za dodatkowe objawy choroby uznaje się także: nadmierną ruchliwość stawów (67% chorych), płaskostopie (71% chorych), wypadanie zastawki mitralnej, a także makroorchidyzm (70% chorych) pojawiający się u chłopców po okresie dojrzewania [25].

## 4 Inne choroby *FMR1*-zależne

Zespół drżenia i ataksji związany z zespołem FXS został opisany po raz pierwszy w 2001 r. u pięciu dziadków, którzy mieli wnuki z potwierdzonymi badaniami molekularnymi zespołem łamliwego chromosomu X [26]. W przeciwieństwie do zespołu FXS bezpośrednią przyczyną wystąpienia objawów FXTAS nie jest brak białka FMRP. Zgodnie z najnowszymi badaniami, wystąpienie choroby związane jest z podwyższoną ekspresją mRNA dla genu *FMR1*, co ma toksyczny efekt względem komórek nerwowych. Wykazano, że cząsteczki mRNA zawierające premutację gromadzą się w dużej ilości w astrocytach i neuronach powodując ich przedwczesne obumieranie, czego efektem jest wystąpienie objawów klinicznych choroby [27]. Badania prowadzone na modelu mysim, wykazały, że myszy – nosicielki premutacji (70-135 powtórzeń CGG) mają podwyższony poziom mRNA genu *FMR1* w mózgach (od 2-3,5-krotnie) [28]. Ponadto badania prowadzone u mężczyzn będących nosicielami wykazały zależność pomiędzy liczbą powtórzeń CGG a aktywnością transkrypcyjną genu *FMR1*. Aktywność ta była 2 i 10-krotnie wyższa u mężczyzn mających odpowiednio powyżej i poniżej 100 powtórzeń sekwencji CGG [29].

Jak wspomniano wyżej zespół drżenia i ataksji związany z zespołem łamliwego chromosomu X został po raz pierwszy opisany w 2001 przez doktor Randi Hagerman. Jest to choroba neurodegeneracyjna wieku dorosłego (objawy w wieku 50-60 lat). U pacjentów obserwuje się głównie drżenie (u 50% pacjentów jest ono łagodne, a u około 17% umiarkowane), problemy z poruszaniem się nasilające się z wiekiem (57%) oraz postępujący deficyt poznawczy i neuropsychologiczny (np. utrata pamięci, niepokój, chwiejność nastroju czy apatia). Ponadto mogą występować u nich dodatkowe objawy, takie jak neuropatia obwodowa (60%), impotencja (80%), zaburzenia czynności pęcherza moczowego i jelit (30-55%). Jak podają dane literaturowe, ryzyko rozwoju choroby uzależnione jest od wielkości premutacji -u osób posiadających allele zawierające <70 powtórzeń CGG jest ono niższe niż u osób o większej liczbie powtórzeń [24].

Nosicielstwo premutacji w genie *FMR1* jest również istotnym czynnikiem wystąpienia przedwczesnego wygasania funkcji jajników związanego z zespołem łamliwego chromosomu X, definiowanego jako spontaniczna menopauza występująca u kobiet przed ukończeniem 40 roku życia, która obejmuje szereg dysfunkcji jajników. FXPOI występuje u ok. 16-20% kobiet, będących nosicielkami premutacji w genie *FMR1* i po raz pierwszy informacja o tej zależności została opisana w 1994 roku przez Schwartza i współpracowników. Ich badania wykazały, że kobiety z premutacją miały wyższe ryzyko wystąpienia nieregularnych miesiączek niż kobiety z allelami z zakresu prawidłowego i z pełną mutacją [30].

Poza tym wykazano, że u kobiet będących nosicielkami premutacji dysfunkcja jajników zależna jest od liczby powtórzeń CGG, jednak związek ten nie jest liniowy. U kobiet o średniej liczbie powtórzeń CGG (80-100 CGG) FXPOI pojawia się częściej (32%) i wcześniej w stosunku do grup nosicielek o mniejszej niż 80 i większej niż 100 liczbie powtórzeń CGG. Przypuszcza się, że u zdrowych kobiet FMRP może odgrywać rolę w aktywacji pęcherzyków i starzeniu się jajników. U nosicielek premutacji gromadzący się mRNA może wywierać toksyczny efekt na funkcjonowanie komórek jajowych. Teoria ta sprawdzana jest w badaniach prowadzonych na pęcherzykach pobranych z jajników samic myszy z premutacją. Wstępne obserwacje wskazują na wysokie stężenie FMRP w komórkach jajowych podczas folikulogenezy. Być może allele z zakresu średniej premutacji warunkują powstawanie największej ilości mRNA białka FMRP w jajnikach, a tym samym generują największą toksyczność, która zaburza ten proces. Sugeruje się również, że w zależności od liczby powtórzeń CGG mRNA mogą przybierać różne konformację np. spinki do włosów, co może wpływać na oddziaływanie z różnymi białkami i cząsteczkami mRNA [5].

## 5 Epidemiologia zespołu łamliwego chromosomu X i chorób *FMR1*-zależnych

Jak wspomniano wcześniej, częstość występowania zespołu łamliwego chromosomu X szacowana jest na 1/4000 u chłopców i 1/5000-8000 u dziewczynek, co jednak nie jest wartością dokładną. Stąd też w celu jednoznacznej oceny częstości występowania zespołu łamliwego chromosomu X oraz innych chorób *FMR1-* zależnych (FXTAS i FXPOI), w wielu krajach podejmuje się próby jej oceny poprzez badania przesiewowe noworodków [31]. Przykładem jest amerykańskie badanie ,,Georgia Public Health Laboratory Newborn Screening Program for *FMR1* DNA methylation’’, w ramach którego, w okresie od kwietnia 2006 do września 2008 roku przebadano ok. 36 tysięcy noworodków płci męskiej. Stwierdzono, że częstość występowania FXS w badanej populacji wynosi 1 na 5161 mężczyzn [32].

Podobne badanie przesiewowe prowadzone było w Kanadzie, jednak badano nie tylko noworodki, lecz również ich matki, co umożliwiło, poza oceną częstości występowania choroby, analizę stabilności przekazywania alleli o różnej długości. W trakcie badania przeanalizowano próbki DNA od 24449 anonimowych par matka - dziecko. Mutacja dynamiczna u noworodków badana była wtedy, gdy u matki stwierdzono obecność allelu zawierającego powyżej 45 powtórzeń CGG. W ten sposób zidentyfikowano 2 chłopców z pełną mutacją, których matki były nosicielkami alleli zawierających 80 i 100 powtórzeń CGG. Na tej podstawie częstość występowania mutacji u chłopców oszacowano na 1/6078 urodzeń [33]. W Europie tego typu badania przeprowadzono m.in. w Anglii i Walii oraz Hiszpanii szacując średnią częstość występowania zespołu FXS odpowiednio na 1/4425 i 1/2633-2466 chłopców [34].

Dzięki badaniom epidemiologicznym udało się także określić częstość występowania premutacji (obecność alleli o 55-200 powtórzeniach CGG, osoby, które posiadają allele w tym zakresie są nosicielami) w populacji ogólnej, która wynosi 1/130-200 u kobiet oraz 1/250-450 u mężczyzn. Obecność premutacji w genie *FMR1* nie wiąże się z występowaniem objawów klinicznych typowych dla zespołu łamliwego chromosomu X, w tym niepełnosprawności intelektualnej. Nie zmienia to jednak faktu, że u części nosicieli choroby obserwuje się zaburzenia psychologiczne, takie jak: problemy z koncentracją i uczeniem się, nadpobudliwość, opóźnienie rozwoju czy problemy emocjonalne. Ponadto stwierdzono, że u ok. 40% mężczyzn i 16% kobiet w starszym wieku, będących nosicielami premutacji, występuje zespół drżenia i ataksji związany z FXS. U około 16-20% kobiet z premutacją występuje przedwczesne wygasanie funkcji jajników związane z zespołem łamliwego chromosomu X [24, 35, 36].

## 6 Podsumowanie

Wczesne rozpoznanie choroby jest kluczowe dla podjęcia odpowiedniego leczenia objawowego i rehabilitacji. Umożliwia także objęcie rodziny chorego dziecka właściwą opieka medyczną, w tym poradnictwem genetycznym. Coraz częściej podejmowane są próby opracowania skutecznej terapii, która miałaby zminimalizować skutki niedoboru białka FMRP w komórkach nerwowych, a tym samym złagodzić objawy kliniczne u pacjentów. W profilaktyce problemów z płodnością ważna jest identyfikacja pacjentek z zespołem przedwczesnego wygasania funkcji jajników związanego z zespołem łamliwego chromosomu X, gdyż obecność premutacji w genie *FMR1* wiąże się ze zwiększonym ryzykiem dziecka chorego na FXS.

Warto wspomnieć, że diagnostyka w kierunku zespołu łamliwego chromosomu X jest jednym z częściej zlecanych badań diagnostycznych nie tylko u dzieci z NI, lecz również u pacjentów z opóźnieniem rozwoju psychoruchowego, opóźnionym rozwojem mowy czy zaburzeniami zachowania, w tym zaburzeniami ze spektrum autyzmu. Zdarzają się pacjenci, u których zostaje potwierdzone rozpoznanie tego zespołu, jeszcze przed rozwinięciem pełnego obrazu klinicznego choroby. Szczególnie istotne jest przeprowadzenie badania w rodzinach, w których niepełnosprawność intelektualna występuje głównie u mężczyzn, w kilku pokoleniach, co wskazuje na defekt genetyczny dziedziczący się w sposób sprzężony z płcią (z chromosomem X). Wykluczenie zespołu FXS powinno być wstępem do poszukiwania defektów w innych genach zlokalizowanych na chromosomie X, których mutacje punktowe lub delecje/duplikacje mogą być przyczyną wystąpienia NI. Tego typu badania, wykorzystujące analizę sprzężeń, sekwencjonowanie metodą Sangera, technikę MLPA czy wreszcie techniki wysokoprzepustowe takie jak porównawcza hybrydyzacja genomowa do mikromacierzy czy sekwencjonowanie następne generacji są również prowadzone w Zakładzie Genetyki Medycznej Instytutu Matki i Dziecka. Badania wykonywane są m.in. w ramach projektów badawczych finansowanych ze źródeł zewnętrznych i umożliwiły identyfikację defektu molekularnego oraz określenie ryzyka ponownego wystąpienia choroby w kilkudziesięciu rodzinach [37, 38, 39].

### Podziękowania

*Pragniemy serdecznie podziękować Pracownikom Zakładu Genetyki Medycznej IMiD, którzy uczestniczyli w diagnostyce i badaniach naukowych nad zespołem łamliwego chromosomu X oraz innych zaburzeń intelektualnych o podłożu genetycznym, którzy nie są współautorami tej pracy*.
